# Hypertension and Atrial Fibrillation: Bridging the Gap Between Mechanisms, Risk, and Therapy

**DOI:** 10.3390/medicina61020362

**Published:** 2025-02-19

**Authors:** Ibrahim Antoun, Georgia R. Layton, Ali Nizam, Joseph Barker, Ahmed Abdelrazik, Mahmoud Eldesouky, Abdulmalik Koya, Edward Y. M. Lau, Mustafa Zakkar, Riyaz Somani, Ghulam André Ng

**Affiliations:** 1 Department of Cardiology, University Hospitals of Leicester NHS Trust, Glenfield Hospital, Leicester LE3 9QP, UK; ali.h.nizam@gmail.com (A.N.); ahmed.abdelrazik@leicester.ac.uk (A.A.); mie7@leicester.ac.uk (M.E.); aik8@leicester.ac.uk (A.K.); el203@leicester.ac.uk (E.Y.M.L.); riyaz.somani@uhl-tr.nhs.uk (R.S.); 2 Department of Cardiovascular Sciences, Clinical Science Wing, University of Leicester, Glenfield Hospital, Leicester LE3 9QP, UKmustafazakkar@me.com (M.Z.); 3 Department of Cardiac Surgery, University Hospitals of Leicester NHS Trust, Glenfield Hospital, Leicester LE3 9QP, UK; 4 National Heart and Lung Institute, Imperial College London, London SW7 2AZ, UK; joseph.barker@nhs.net; 5 National Institute for Health Research, Leicester Research Biomedical Centre, Leicester LE3 9QP, UK

**Keywords:** atrial fibrillation, hypertension, mechanism, review

## Abstract

*Background and objectives:* Atrial fibrillation (AF), the most prevalent sustained arrhythmia, poses a significant public health challenge due to its links with stroke, heart failure, and mortality. Hypertension, a primary modifiable cardiovascular risk factor, is a well-established risk factor for AF that facilitates structural and electrical changes in the atria, including dilation, fibrosis, and pressure overload. *Material and Methods:* we conducted a literature search regarding the shared mechanisms, risks and treatments of hypertension and atrial fibrillation. *Results:* The renin–angiotensin–aldosterone system plays a pivotal role in this remodelling and inflammation, increasing AF susceptibility. Uncontrolled hypertension complicates AF management, diminishing the effectiveness of mainstay treatments, including antiarrhythmic drugs, catheter ablation, and cardioversion. Effective blood pressure management, particularly with therapies targeting the renin–angiotensin–aldosterone system (RAAS), can lower the risk of new-onset AF and reduce the incidence of recurrent AF, enhancing the success of rhythm control strategies. These antihypertensive therapies mitigate myocardial hypertrophy and fibrosis and attenuate both atrial pressure strain and the inflammatory response, mitigating the substrates for AF. *Conclusion:* This review highlights the urgent need for integrated strategies that combine BP control, AF screening, and lifestyle modifications to minimise the burden of AF and its complications. Future research should investigate the specific mechanisms of cellular-level interactions associated with a hypertensive predisposition to AF, including systematic inflammation and the role of genetics, the impact of blood pressure variations on AF risk, and individualised treatment strategies specifically targeting the shared mechanisms, simultaneously propagating hypertension and AF.

## 1. Introduction

Atrial fibrillation (AF) is estimated to affect 2–4% of our global adult population, but its prevalence is expected to more than double as life expectancy increases and undiagnosed cases are more frequently detected. While ageing is a key risk factor for AF, other conditions like hypertension, diabetes, heart failure, coronary artery disease, chronic kidney disease, obesity, sleep apnoea and invasive cardiac procedures also play a major role. Many of these risk factors are modifiable, making lifestyle changes and early intervention important in preventing AF onset, slowing its progression and minimising its consequences on quality of life. Women and non-Caucasian individuals generally have lower rates of AF compared to men and Caucasians, but the lifetime risk remains significant. Recent estimates suggest that one in three people of European ancestry will develop AF by the age of 55. This shows how critical it is to address risk factors early to reduce the growing burden of AF.

AF represents a significant public health burden due to its associated risks, including stroke, impaired quality of life, readmissions, heart failure, and mortality [1,2,3,4,5,6,7,8], and its prevalence now is similar to that of myocardial infarction and greater than other major cardiovascular comorbidities, including cerebrovascular disease and heart failure [9]. Hypertension is the leading modifiable risk factor for cardiovascular disease morbidity and mortality globally [10], and it is estimated that more than 40% of patients affected may not yet have been identified. It has been consistently recognised as a major contributor to the prevalence and progression of AF. Epidemiological studies reveal that hypertension increases the risk of AF by approximately 50%, with the degree of risk closely linked to the severity, variability, and duration of elevated blood pressure (BP) [11]. AF and hypertension share multiple risk factors, as shown in Table 1.

The pathophysiological mechanisms underlying the relationship between hypertension and AF are multifaceted, encompassing the structural, biochemical, and electrical remodelling of the heart. Prolonged hypertension promotes left atrial dilation, fibrosis, and pressure overload, creating a substrate that predisposes the myocardium to arrhythmogenic activity [12]. The renin–angiotensin–aldosterone system (RAAS) has emerged as a pivotal mediator in this interplay, with its activation contributing to both atrial remodelling and inflammation, further perpetuating AF and mitigating the impact of anti-AF treatments [13]. Effective BP management is crucial in reducing AF’s incidence and recurrence. Evidence indicates that antihypertensive therapies aimed at the RAAS lower BP and help alleviate atrial remodelling, underscoring their dual benefits in preventing AF. Furthermore, innovative interventional strategies, such as renal sympathetic denervation, show promise in concurrently tackling hypertension and AF [14].

This article examines the intricate relationship between hypertension and AF, concentrating on epidemiology, pathophysiology, and clinical implications. By elucidating the mechanisms connecting these two conditions, we aim to emphasise the significance of integrated management strategies in mitigating AF risk, enhancing treatment outcomes, and alleviating the burden of cardiovascular disease. This review provides an up-to-date overview of the current evidence linking hypertension with the onset and propagation of atrial fibrillation.

## 2. Hypertension and AF Risk

Epidemiological data indicate that hypertension doubles the risk of AF, with the level of risk being proportional to the increasing severity and duration of elevated blood pressure. For instance, the Framingham Heart Study identified hypertension as an independent cause of AF, attributing one in five cases of arrhythmia to high blood pressure [11]. Furthermore, a systematic review and meta-analysis by Aune et al. highlighted that higher systolic and diastolic BP positively correlate with the risk of AF, reinforcing the notion that hypertension is a critical risk factor for arrhythmia [15]. Various factors, including the level, variability, and duration of elevated BP, influence the risk of AF in hypertensive individuals. Higher systolic and diastolic pressures correlate with a greater likelihood of AF, and isolated systolic hypertension is particularly important in older adults [16]. Also, a recent meta-analysis has shown that high systolic and diastolic BP are associated with an increased risk of AF, particularly in older populations [15]. BP variability, assessed through ambulatory BP monitoring (ABPM), has also emerged as an independent predictor of AF risk [17]. Although not extensively studied, it is believed to be caused, at least in part, by increased arterial stiffness and autonomic nervous system dysregulation. It is, therefore, commonly seen in patients with diabetes. It may also be associated with poor patient compliance with blood pressure therapy or lifestyle factors such as high alcohol intake. BP variability has been independently demonstrated to lead to a reduction in the atrial effective refractory period because of LV remodelling and pulmonary vein dilation. Additionally, it is associated with arterial stiffness and remodelling, which were predictive of incident AF in the Framingham heart study [18]. In addition, it is also hypothesised to cause endothelial cell dysfunction secondary to impaired nitrous oxide formation and autonomic neuropathy through inappropriate RAAS activation [19].

Furthermore, the duration of hypertension plays a critical role, with prolonged exposure to elevated BP exacerbating cardiovascular remodelling and increasing AF susceptibility [17]. Certain hypertensive subgroups face an amplified risk of AF. Older adults, who often experience vascular stiffening and other comorbidities, are particularly vulnerable [20]. Similarly, hypertensive individuals with targeted organ damage, such as left ventricular hypertrophy or microvascular dysfunction, have a higher likelihood of developing AF [21]. Additionally, the coexistence of other risk factors—such as obesity, diabetes, and obstructive sleep apnoea—further increases the probability of AF in hypertensive populations [22]. This is because there is a temporal relationship between AF and HTN, where not only the presence of hypertension but the duration for which it affects a patient and is untreated or uncontrolled plays a critical role in both the development and persistence of AF. This is not surprising given that long-standing hypertension results in structural and functional end-organ damage, both within the heart itself and in associated organ systems, resulting in left ventricular hypertrophy (LVH), diastolic dysfunction, chronic kidney disease (CKD), and cerebrovascular disease.

Additionally, the presence of hypertension is often accompanied by other cardiovascular risk factors, such as obesity and diabetes, which further complicate the clinical picture and increase the likelihood of AF [23]. The strong association between hypertension and AF underscores the importance of comprehensive risk-reduction strategies. Proactive screening for AF in patients with long-standing or poorly controlled hypertension is vital, particularly in high-risk groups. BP control remains a cornerstone of AF prevention, with evidence suggesting that antihypertensive agents targeting the renin–angiotensin system may provide additional benefits by reducing atrial remodelling and inflammation [24]. Lifestyle modifications, including weight loss and the treatment of comorbid conditions such as sleep apnoea, further enhance risk reduction. Similarly, Emdin et al. found that a 20 mmHg increase in systolic BP was associated with a 23% increase in the risk of developing AF, further emphasising the impact of BP on AF prevalence [25]. This relationship is observed in general populations and specific demographics, such as elderly patients. Koffi et al. reported that individuals with a pulse pressure greater than 65 mmHg had a significantly higher risk of AF [26]. Findings from various cohort studies further support hypertension’s role in AF. For instance, Mohanty et al. noted that hypertensive patients have a 14% increased risk of developing AF, which aligns with the broader understanding that the hemodynamic changes associated with hypertension, such as left ventricular hypertrophy and increased left atrial pressure, contribute to the pathophysiology of AF [27]. Moreover, studies have indicated that even high–normal BPs can predict the onset of AF, as shown by Grundvold et al., who identified upper–normal BP as a risk factor for incident AF in middle-aged men [28].

The relationship between HTN and AF is proposed to be bi-directional. Emerging evidence suggests atrial remodelling and endothelial dysfunction may be central mechanisms linking HTN, AF, stroke, and heart failure [29]. Endothelial dysfunction impairs nitric oxide bioavailability, promoting inflammation, oxidative stress, and vascular stiffness, all contributing to atrial fibrosis and impaired myocardial function [30]. Atrial remodelling is a process of cell death, including the hypertrophy of myocytes, the proliferation of fibroblasts, and the laying down of the excess extracellar matrix, leading to fibrosis. This reactive fibrosis increases the distance between neighbouring myocyte bundles, disrupting electrical continuity and delaying conduction [31]. This exacerbates hemodynamic disturbances and creates a substrate for arrhythmogenesis, solidifying AF as a marker and mediator of systemic cardiovascular pathology [32]. Understanding this interconnected pathophysiology underscores the importance of the early detection and comprehensive management of HTN to mitigate downstream complications, including AF, stroke, and heart failure.

In summary, HTN is a major modifiable risk factor for atrial fibrillation, with its severity and duration directly influencing AF development. The early detection and control of both blood pressure and other modifiable risk factors are essential to preventing atrial remodelling and the resultant permanent atrial fibrillation that occurs as a result. Therefore, primary prophylaxis should be preferred. Comprehensive management strategies are critical to mitigate long-term risks from AF and improve cardiovascular outcomes. Management approaches are multifaceted and individualised to the patient, given the broad range of mechanisms through which HTN contributes to AF onset. These are discussed in detail below.

## 3. Pathophysiology of AF in Hypertension

HTN and AF are closely linked through several pathophysiological mechanisms, including structural remodelling, haemodynamic stress, neuroendocrine activation, inflammation, and changes to the autonomic nervous system. The pathophysiological mechanisms linking hypertension and AF include the structural and electrical remodelling of the heart. Pressure loading from elevated BP leads to increased left atrial volume through dilation and pressure through wall thickening and stiffening, causing dilation, fibrosis, and electrical conduction abnormalities, facilitating the development of AF [33,34].

**Figure 1:**
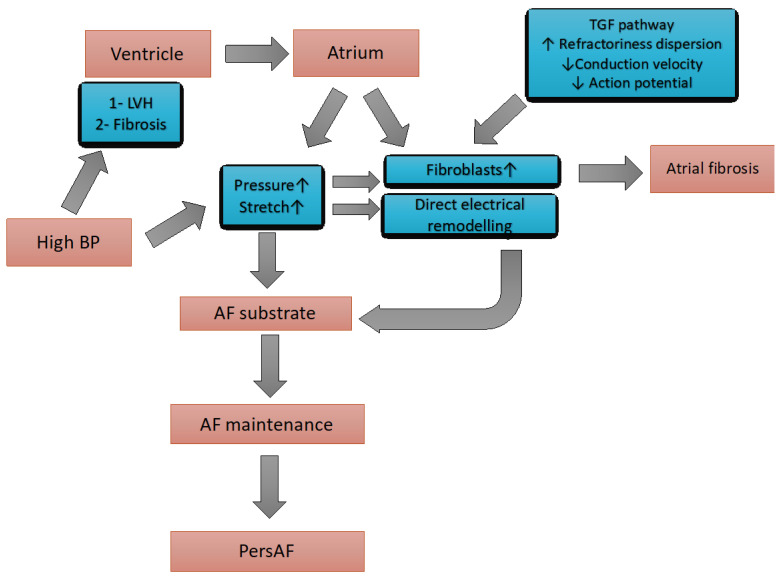
Mechanism of atrial fibrillation in hypertension patients. AF: atrial fibrillation. BP: blood pressure. persAF: persistent atrial fibrillation. LVH: left ventricular hypertrophy. ↓: decrease. ↑: increase. TGF: transforming growth hormone.

Several animal models have been developed to investigate the pathophysiological mechanisms underlying the increased propensity of hearts in animals or humans with hypertension that develop AF. Experimental models of hypertension included partial stenosis of the renal artery stenosis [35], ascending aorta [36], or exposure to corticosteroids [37]. Other studies employed spontaneously hypertensive rats (SHRs) matched with controls [38,39]. Some of these investigations concentrated on detecting structural and functional abnormalities of the LA that are considered potential triggers of AF. Altered calcium handling by the atrial myocytes has been noted as a mechanism potentially promoting AF. Pluteanu et al. demonstrated the existence of subcellular alterations in calcium handling in SHRs, which were correlated with an increased propensity of atrial myocytes to develop frequency-dependent, arrhythmogenic calcium alternans [40]. Other arrhythmogenic mechanisms seem to be involved. In SHRs, the ultrastructure observation of myocytes showed enhanced neoformation of side-to-side gap junctions, reducing end-to-end-type junctions [41]. These changes, which can increase the propensity to tachyarrhythmias [42], are overexpressed in rats with hypertension induced by stenosis of the ascending aorta [36]. Additional research has encompassed electrophysiological studies of the LA, typically assessing the conduction velocity and inducibility of AF through various techniques. In humans, several mechanisms may be involved in the genesis of AF in hypertensive patients. A central role is expressed by the so-called atrial cardiomyopathy, a complex of structural, architectural, contractile, and electrophysiological changes affecting the atria with the potential to produce clinically relevant manifestations [43], predominantly hemodynamic and nonhemodynamic mechanisms (Figure 1).

The predominantly hemodynamic mechanisms include the increased left ventricular (LV) wall thickness, increased LV stiffness, and impaired LV diastolic function associated with hypertension. These processes may lead to a rise in LA stretch and pressure, with subsequent remodelling and dysfunction of the LA, ultimately predisposing to AF. Several experiences in animals and humans support the role of histological changes in the atria as potential triggers of AF [44]. The most important atrial changes include fibroblast proliferation, extracellular matrix alterations, and myocyte hypertrophy [44]. The resulting disorders of interconnections between muscle bundles may lead to the shortening of LA refractoriness, unidirectional blocks, and re-entry phenomena. These processes may initiate AF, eventually triggered by ectopic stimuli from pulmonary veins or other sites. A European Consensus reviewed the complex interplay between cardiac arrhythmias and hypertension [45].

The atria of hypertensive patients may show subtle electrophysiological changes that predispose them to AF. In a detailed electrophysiological study involving hypertensive patients and normotensive controls, the former group exhibited the slowing down of both global and regional conduction, an increase in areas of low voltage, and, importantly, the higher inducibility of sustained AF [12]. These changes may occur due to the remodelling of the wall through physical stretching but not due to wall thinning. The Framingham study was the first to establish a link between BP and LA dilation, indicating a positive association with increasing systolic BP, but not diastolic or pulse pressures, with larger atrial size, independent of age and patient BMI [18]. Another Framingham report noted that the risk of AF rose with the increasing LA diameter and LV wall thickness whilst being inversely related to the shortening fraction [46]. In a study involving a large cohort of initially untreated hypertensive subjects, baseline LV hypertrophy almost doubled the risk of new-onset AF. For each one-standard-deviation increase in LV mass, the risk of AF increased by 20%. Regardless of the LV mass level, the 5-year risk of permanent AF heightened with LA diameter [47]. Hence, in hypertensive patients with a sinus rhythm at entry and without other predisposing conditions, the risk of new-onset AF grew with both age and LV mass in this study. Similarly, LV hypertrophy, detected via electrocardiography, is a key predictor of AF in the general population, as well as hypertensive individuals [48,49]. A larger LA size is more strongly associated with the onset of permanent, rather than transient, AF. This logically suggests that the atria become more resistant to commonly used antiarrhythmic interventions once remodelling has occurred. Additionally, other research has indicated that LA dilatation, linked with atrial pump function and LV diastolic dysfunction, identifies patients at a heightened risk of AF [50,51].

Several systemic patient factors exacerbate AF through the propagation of atrial remodelling, electrolyte imbalance, inflammation, or electrical instability. Many are driven by complicated hypertension and the end-organ damage that develops with prolonged uncontrolled high blood pressure. These so-called “extra-pulmonary” triggers act independently or synergistically with hypertension-induced structural changes to promote AF onset and persistence. Key extra-pulmonary triggers include inflammatory states, external triggers such as surgery, apnoeic disorders, and renal and autonomic nervous system disorders. These are considered individually below.

## 4. The Role of Inflammation

Patients without pre-existing AF who undergo invasive procedures are at risk of new-onset AF (NOAF). Although more likely to be transient than AF associated with a hypertensive trigger, it remains a negative predictor of long-term morbidity and mortality [52]. Defined as “new AF associated with a precipitating and potentially reversible factor”, this patient group are affected by “trigger-induced” AF. Common triggers peri-operatively may include physiological stress, electrolyte abnormalities, autonomic dysregulation and acute inflammatory states. Sometimes impacting patients who have not undergone procedures, other common triggers may include sepsis, drugs, and inflammatory conditions such as endocrine disorders, primary diseases of the heart, including myocarditis or pericarditis, and obstructive sleep apnoea (OSA) [53].

Many of these conditions, which independently elevate the risk of NOAF, show elevated levels of circulating cytokines that play key roles in the acute inflammatory response. Common to many of these conditions are interleukin-6 (IL-6), tumour necrosis factor-a (TNF-a) and the C-reactive protein (CRP), with the potential for these cytokines to function as predictive biomarkers of patients at risk of AF [54].

IL-6 and TNF-a are inflammatory markers linked directly and indirectly to both hypertension and AF. IL-6 may contribute to the initiation, progression, and maintenance of hypertension by reducing nitric oxide bioavailability, increasing vascular oxidative stress, regulating angiotensin II expression, and altering vascular function and structure [55].

CRP is a marker of systemic inflammation, often elevated in hypertension, and may contribute to vascular dysfunction, with elevated levels of both IL-6 and CRP found in patients with dilated left atria [56]. IL-6 is a major stimulus for hepatic CRP production, so associations with CRP in this regard are likely due to a preceding IL-6 elevation only rather than an independent effect. TNF-a is associated with the structural remodelling of the atrium, specifically the angiotensin-II-dependent histological changes seen in hypertension [57]. A direct association of these cytokines, especially IL-6 and TNF-α, can be demonstrated with atrial fibrosis progression, facilitating AF development. These markers play a role in both the initiation and progression of hypertension and AF, with inflammation potentially serving as a common pathway in both conditions. Suppressing inflammation, therefore, is a common mechanism by which some antihypertensive therapies can exert their effect either directly or as an indirect consequence of their intended purpose.

Angiotensin-converting enzyme inhibitors (ACEIs) and angiotensin receptor blockers (ARBs) reduce the secretion of pro-inflammatory cytokines such as IL-6 and TNF-α and lower the production of reactive oxygen spices from the angiotensin II signalling pathway [58]. Aldosterone promotes inflammation by stimulating cytokines such as IL-1β and ROS. The mineralcorticoid receptor antagonists (MRAs) spironnalactone and eplerenone counteract these effects by blocking aldosterone’s receptor and reducing activity in the NF-κB pathway, which is a key regulator of inflammation, further decreasing inflammatory signalling and limiting atrial remodelling [59]. Additional therapies, not directly used for hypertension but its associated conditions, can also modulate this inflammatory response. Statins exert anti-inflammatory effects through their inhibition of the HMG-CoA reductase enzyme, decreasing levels of cytokines, including IL-6 and TNF-α, whilst improving endothelial function through a reduction in oxidative stress and improved nitric oxide bioavailability, thus protecting against some inflammation-driven atrial remodelling [60].

Specific anti-inflammatories have been trialled to reduce AF. Colchicine, the long-used anti-gout drug, inhibits IL-1β-induced IL-6 release and subsequent atrial fibrosis. Although studies have demonstrated mixed results, with some not finding benefits, they demonstrate moderate benefits in reducing AF, particularly in patients undergoing cardiac surgery or procedures such as PVI [61]. Similarly, the direct suppression of IL-1β with specific monoclonal antibody canakinumab results in the downstream suppression of IL-6 and TNF-α, limiting atrial remodelling and making it an excellent potential therapy to reduce AF burden. Although randomised studies of this drug suggest a reduction in AF burden, it has been associated with increased rates of infection-related mortality. It is not routinely used for the treatment of AF [62].

## 5. Special Patient Groups: Patients Requiring Invasive Cardiac Procedures and Surgery

Advancing life expectancies and improved post-surgical outcomes have led to older and more comorbid patients undergoing surgical cardiac procedures in recent decades [63]. Therefore, an increasing proportion of this cohort is known to have AF before intervention. Untreated pre-operative AF is associated with increased morbidity and mortality, especially after open heart surgery and transcatheter valve procedures [64]. Randomised data have demonstrated no increase in morbidity or mortality when considering surgical ablation with or without surgical left atrial appendage occlusion at the time of concurrent cardiac surgeries. This strategy increases freedom from long-term AF and its consequences, particularly in conditions strongly associated with left atrial enlargement and AF, such as mitral valve disease [65]. Therefore, international guidelines recommend that patients with AF undergoing cardiac surgery undergo surgery for AF simultaneously, such as the maze procedure, if not contra-indicated [53]. Furthermore, surgical or hybrid ablation can be considered a stand-alone procedure for patients where less invasive options have failed to treat their arrhythmia.

## 6. Special Patient Groups: Obstructive Sleep Apnoea

OSA may contribute to the pathogenesis of AF in hypertensive individuals. Clinical studies indicate that OSA, which affects roughly half the patients with hypertension [66], is related to LA dilatation and an increased risk of AF [67], regardless of the absence or presence of LV dysfunction [68,69]. Many mechanisms by which OSA propagates this arrhythmogenesis are similar to those discussed previously in the review and are associated indirectly with inflammation and atrial remodelling.

## 7. The Renin–Angiotensin–Aldosterone System’s Role in AF Pathogenesis and Maintenance

**Figure 2:**
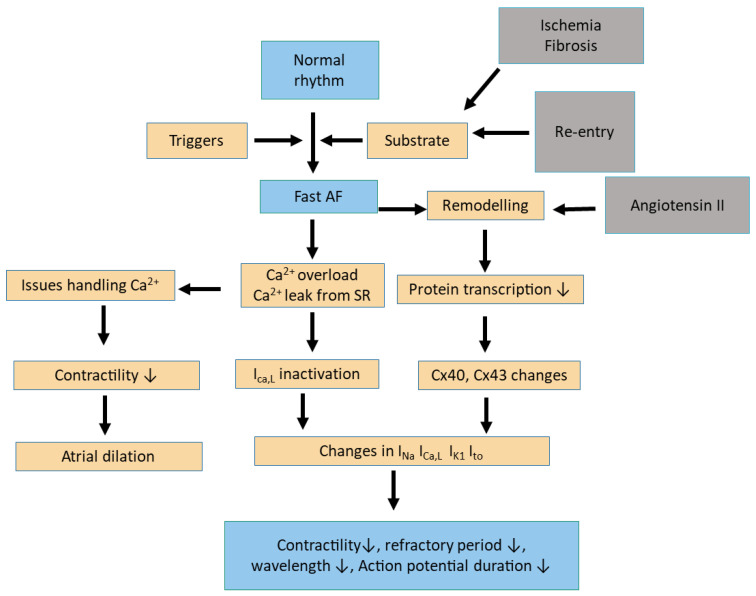
Role of the renin–angiotensin–aldosterone system (RAAS) in atrial fibrillation (AF). Ca^2+^: calcium. AF: atrial fibrillation. SR: sinus rhythm. ↓: decrease.

The renin–angiotensin–aldosterone system (RAAS) plays a pivotal role in AF. The activation of the RAAS has been implicated in the structural and electrical remodelling of the atria, which are critical processes in the development and maintenance of AF. Specifically, angiotensin II, a key effector of the RAAS, contributes to atrial fibrosis and hypertrophy, promoting an arrhythmogenic substrate, as shown in Figure 2 [70,71,72]. Research indicates that RAAS activation increases collagen deposition and cardiomyocyte apoptosis, which are essential in atrial remodelling [70,73,74]. The structural changes associated with AF include atrial dilation and fibrosis, which can disrupt normal electrical conduction and increase the likelihood of arrhythmias [75].

Furthermore, aldosterone, another component of the RAAS, has been shown to induce oxidative stress and inflammation within cardiac tissues, further exacerbating the fibrotic process and contributing to the AF substrate [73,76,77]. It is believed that through aldosterone-mediated receptors, angiotensin II primes an inflammatory response through the selective upregulation of IL-6 [78]. Elevated levels of implicated cytokines such as IL-6 promote gap-junction dysfunction, contributing to atrial electrical remodelling, which primes AF onset [79]. The inhibition of the RAAS through ACEIs and ARBs has demonstrated efficacy in reducing the incidence of AF. Similarly, these agents have been shown to reduce implicated circulating inflammatory mediators such as IL-6 and CRP [80]. These agents mitigate the adverse effects of angiotensin II and improve atrial structural remodelling by reducing fibrosis and hypertrophy [24,81,82]. For instance, studies have shown that using MRAs like spironolactone can attenuate atrial remodelling and decrease the duration of AF episodes in experimental models [81]. Moreover, combining antiarrhythmic drugs with RAAS inhibitors has been suggested to enhance the prevention of AF by modifying the underlying arrhythmic substrate [83,84]. Clinical evidence supports the notion that RAAS modulation can improve outcomes in patients with AF. For example, a systematic review indicated that RAAS inhibitors are associated with a lower incidence of AF in patients with heart failure and hypertension [13,85]. Additionally, using RAAS blockers has been linked to reduced postoperative AF, suggesting their protective role in various clinical settings [82].

## 8. Benefits of BP Control in AF Patients

The management of BP in patients with AF is crucial due to the significant interplay between hypertension and the pathophysiology of AF. Elevated BP is a well-established risk factor for the development and progression of AF, and effective BP control can improve outcomes in these patients. Hypertension contributes to the structural and electrical remodelling of the atria, which is a key factor in the development of AF. The relationship between hypertension and AF is further supported by findings that aggressive BP management can reduce the incidence of AF. For instance, the Cardio-Sis trial demonstrated that patients with tightly controlled systolic BP (<130 mm Hg) had a lower risk of new-onset AF than those with less stringent control [86]. This suggests that maintaining optimal BP levels is essential in preventing AF, particularly in hypertensive patients. Autonomic nervous system dysregulation contributes to hypertension-driven AF. Both sympathetic and parasympathetic activations have been suggested to trigger AF [87]. The sympathetic stimulation of adrenergic receptors stimulates intracellular calcium flux, which promotes the late depolarisation of myocytes. Conversely, the parasympathetic stimulation of muscarinic receptors can trigger potassium flux, shortening the refractory period of the action potential and priming arrhythmias. Animal studies have evaluated this concept by demonstrating improved electrophysiological conditions and reduced AF burden following renal sympathetic denervation [88]. Moreover, the role of the autonomic nervous system in AF is influenced by BP variability. Increased beat-to-beat BP variability, often observed in AF patients, can lead to heightened sympathetic nervous system activity, further exacerbating arrhythmogenesis [89,90]. Therefore, stabilising BP helps reduce the overall burden of AF and mitigates the sympathetic overactivity associated with fluctuating BP levels. Recent interventions such as renal sympathetic denervation (RSD) have shown promise in managing both hypertension and AF. When combined with PVI, RSD has been associated with significant reductions in both the BP and AF burden, particularly in patients with resistant hypertension [91,92]. This dual approach highlights the importance of targeting both hypertension and AF simultaneously to achieve better clinical outcomes.

Additionally, the RAAS plays a critical role in regulating BP and has been implicated in the pathogenesis of AF. Angiotensin II, a key hormone in the RAAS, not only elevates BP but also promotes atrial fibrosis and inflammation, which can lead to AF [93]. Therefore, pharmacological strategies that effectively reduce BP while modulating the RAAS may offer additional benefits in preventing AF. The implications of BP control extend beyond the prevention of AF; they also encompass the management of comorbid conditions that frequently coexist with AF, such as heart failure and coronary artery disease. Effective BP management can improve myocardial perfusion and reduce the risk of adverse cardiovascular events, including stroke, which is a significant concern in AF patients [94,95]. Moreover, the relationship between BP variability and AF has been highlighted, with studies indicating that increased beat-to-beat BP variability is associated with heightened sympathetic nervous system activity, which can further destabilise cardiac rhythm [89]. Therefore, controlling BP in patients with AF is essential for reducing the risk of AF development and recurrence. Effective BP management can mitigate the structural and electrical changes in the atria, reduce sympathetic nervous system activation, and improve overall cardiovascular health. This multifaceted approach underscores the need for integrated management strategies that address hypertension and AF to optimise patient outcomes.

It is evident that there are a variety of associations linking HTN with AF progression and onset and that this relationship varies depending on a patient’s specific hypertension phenotype. Each phenotype is characterised by differing pathophysiology. Persistent high blood pressure is more highly associated with LVH and resulting diastolic dysfunction, leading to chronic LA pressure overload and fibrosis. More labile blood pressure phenotypes result in intermittent pressure surges, triggering acute atrial stretch responses and leading to autonomic dysregulation [96]. Patients with resistant high blood pressure demonstrate heightened sympathetic activity and RAAS overactivation, as well as chronic inflammation [97]. External to these hypertension phenotypes are additional extra-pulmonary triggers, primarily associated with metabolic syndrome and obesity, resulting in epicardial fat accumulation, insulin resistance, and systemic inflammation. Therefore, the management of hypertension in the context of AF requires highly tailored therapy specific to the patient phenotype—such as RAAS inhibitors for fibrosis-prone individuals, β-blockers for those with heightened sympathetic activity, and mineralocorticoid receptor antagonists for resistant hypertension—in order to optimise hypertension-driven AF prevention and address the dominant mechanisms driving arrhythmogenesis within each phenotype.

## 9. The Effect of Hypertension on AF Treatment

The treatment of AF often involves the use of antiarrhythmic drugs and anticoagulants, which the presence of hypertension can influence. Studies indicate that hypertensive patients may experience a higher burden of AF, which can complicate the effectiveness of antiarrhythmic medications. For instance, the presence of hypertension is associated with a greater risk of AF recurrence after treatment, particularly in patients undergoing radiofrequency ablation [98]. Hypertensive patients often exhibit structural changes in the heart, such as left atrial enlargement, which can lead to reduced response to antiarrhythmic therapy [99,100]. Moreover, hypertension can affect the choice of anticoagulants. Patients with uncontrolled hypertension may have an increased risk of bleeding complications when treated with anticoagulants, necessitating the careful monitoring and management of BP levels [98,101]. Consequently, achieving optimal BP control is essential for maximising the effectiveness of anticoagulation therapy in AF patients [102]. The pharmacological management of AF typically involves rate control and rhythm control strategies, often using beta-blockers, calcium channel blockers, and antiarrhythmic drugs. However, the presence of hypertension can complicate these treatment strategies. For instance, patients with uncontrolled hypertension may experience more frequent episodes of AF, leading to a higher burden of symptoms and a greater need for hospitalisation [103]. Additionally, the efficacy of antiarrhythmic medications may be diminished in hypertensive patients due to altered pharmacokinetics and the presence of comorbidities [101].

Catheter ablation has emerged as a prominent treatment option for patients with symptomatic AF, particularly those who are refractory to pharmacological therapy. However, the presence of hypertension can significantly impact the outcomes of catheter ablation procedures. Studies have shown that patients with hypertension are at a higher risk of AF recurrence following ablation compared to their normotensive counterparts. This is attributed to several factors, including the presence of LA enlargement and left ventricular hypertrophy, both of which are common in hypertensive patients and are associated with poorer ablation outcomes [104]. Furthermore, the degree of hypertension may correlate with the complexity of the ablation procedure. For instance, patients with more severe hypertension may exhibit more extensive atrial remodelling, which can complicate the ablation process and increase the likelihood of the incomplete isolation of pulmonary veins, which is a common target in AF ablation [105]. Consequently, the effective management of hypertension before and following ablation may improve procedural outcomes and reduce the risk of AF recurrence [47]. The recurrence of AF after ablation, specifically after pulmonary vein isolation procedures, is common in patients with hypertension and may result in the need for repeat ablation procedures or referral for surgical ablation [106]. Electrical cardioversion is a procedure to restore normal sinus rhythm in patients with AF. The presence of hypertension can also influence the success of cardioversion. Research indicates that hypertensive patients may have a lower success rate for cardioversion compared to those without hypertension. This may be due to the structural changes in the atria associated with chronic hypertension, such as atrial dilation and fibrosis, which can hinder the restoration of normal rhythm. Moreover, the risk of complications during cardioversion, such as thromboembolic events, may be heightened in hypertensive patients, particularly if anticoagulation is not adequately managed. Performing cardioversion in patients with AF who have been in the arrhythmia for more than 48 h poses a risk of thrombus formation in the left atrial appendage, which can lead to stroke if dislodged [107,108]. Therefore, the careful assessment and management of BP before cardioversion are essential to optimise outcomes and minimise risks.

In summary, hypertension significantly impacts the success or failure of AF treatments, including pharmacological management, catheter ablation, and electrical cardioversion. Hypertension is linked to a greater burden of AF, an increased risk of recurrence following ablation, and lower success rates for cardioversion. The effective management of hypertension is vital to improve treatment outcomes for patients with AF. Future research should concentrate on developing integrated treatment strategies that address both hypertension and AF to optimise patient care and reduce the associated morbidity and mortality.

## 10. Future Perspectives

The intricate relationship between hypertension and AF opens a valuable avenue for future research, particularly in uncovering shared mechanisms and optimising prevention and treatment strategies. As atrial remodelling and endothelial dysfunction pathophysiology become better understood, the potential to leverage advanced technologies, including artificial intelligence (AI), is increasingly apparent.

One focus should be on the development of AI-powered predictive models to identify individuals at the highest risk of AF, stroke, and heart failure in hypertensive populations. By integrating clinical, genetic, and imaging data, machine learning algorithms can predict disease progression, stratify risk, and guide personalised interventions. For example, AI-based electrocardiographic analysis has already shown promise in detecting subclinical AF and identifying patterns indicative of underlying atrial remodelling [109,110].

Additionally, AI could facilitate identifying and verifying potential biomarkers for atrial remodelling and endothelial dysfunction, such as IL-6 and TNF-alpha. By analysing large datasets from genomic, proteomic, and metabolomic studies, AI-driven approaches could reveal new therapeutic targets and enhance our understanding of the molecular pathways linking hypertension, AF, and associated complications [111]. Similarly, machine learning could support the development of predictive models for new AF events based on submitted biomarker profiles. This would support personalised approaches to treatment. By analysing patient-specific data, including blood pressure variability, medication responses, and lifestyle factors in combination with biomarker profiles, such approaches could improve the efficacy of intervention by optimising currently available RAAS modulating treatments and lifestyle modifications while minimising adverse outcomes.

Furthermore, incorporating AI into large-scale clinical trials could transform research methods. AI-powered adaptive trial designs could allow for real-time adjustments to protocols based on patient responses, thereby accelerating the evaluation of emerging therapies and targeting shared mechanisms of hypertension and AF. Finally, AI-integrated wearable devices hold significant potential for continuously monitoring blood pressure and arrhythmias. These tools can provide real-time feedback to patients and clinicians, enabling the early detection of atrial remodelling and preventing complications through timely interventions [112]. Future work can combine advanced AI technologies with ongoing research into the shared pathophysiology of hypertension and AF to develop more precise, personalised, and effective strategies to mitigate the global burden of these intertwined conditions.

Despite its potential in both risk prediction and treatment personalisation, there are currently major barriers preventing AI’s widespread adoption within modern cardiology care. AI models require large-scale, validated datasets, but most of our clinical data are small, fragmented and subject to significant biases due to the heterogeneity of electronic health records and imaging modalities. Once this can be overcome to allow AI workflows to be used in routine practice, they must integrate into our current healthcare systems without adding excessive complexity; many modern healthcare systems currently lack the infrastructure to support real-time AI-driven decision making. Perhaps most importantly, the use of AI for any healthcare purpose raises concerns about data privacy and security, as well as the potential for the introduction of bias into the algorithms, given that bias lies within most of our currently available datasets that could be used to train these models, posing the risk of training models that exacerbate current healthcare disparities, rather than improving them. Implementing AI into modern hypertension treatment will require the development of standardised frameworks and regulatory pathways that currently do not exist, and it holds great potential for enhancing early detection and risk mitigation for patients with both hypertension and AF.

## 11. Conclusions

Hypertension is a key driver of AF pathogenesis, significantly contributing to the global cardiovascular disease burden. The strong epidemiological link between hypertension and AF highlights the importance of effective blood pressure management in reducing AF risk. Chronic hypertension promotes atrial structural and electrical remodelling, primed by inflammatory cytokines, creating a substrate for AF initiation and persistence. Additionally, elevated blood pressure reduces the efficacy of AF treatments, including antiarrhythmic drugs, catheter ablation, and electrical cardioversion, increasing recurrence rates.

The renin–angiotensin–aldosterone system (RAAS) plays a central role in this interplay, driving atrial remodelling through fibrosis, hypertrophy, and inflammation. Targeting the RAAS with inhibitors such as ACEs and ARBs can mitigate inflammation, reduce AF incidence, and improve treatment outcomes. Blood pressure control in AF patients also lowers the risk of complications, including stroke, heart failure, and other cardiovascular events. Given the interconnected nature of hypertension and AF, an integrated management strategy is essential. This should include optimal blood pressure control, early AF screening in high-risk hypertensive individuals, and lifestyle modifications targeting shared risk factors such as obesity, obstructive sleep apnoea, and diabetes. Emerging therapies, such as renal sympathetic denervation, may offer dual benefits by addressing both conditions simultaneously.

Artificial intelligence (AI) holds promise in revolutionising AF and hypertension management by enabling earlier detection, risk stratification, and personalised treatment. AI-driven algorithms can analyse large datasets from wearable devices, electronic health records, and imaging studies to identify high-risk individuals, predict AF episodes, and optimise blood pressure control strategies. Machine learning models may also enhance decision making in AF management by tailoring treatment based on patient-specific hemodynamic and genetic profiles. Future research should focus on integrating AI-based predictive models into clinical practice to improve prevention, treatment, and long-term outcomes.

In conclusion, hypertension not only contributes to AF pathogenesis but also influences treatment success. Targeting hypertension as a primary therapeutic approach in AF management can significantly reduce the arrhythmia burden and improve long-term cardiovascular outcomes. Future research should explore personalised and integrated strategies, incorporating AI-driven approaches to optimise risk assessment, early intervention, and individualised treatment in this high-risk population.

## Figures and Tables

**Table 1:** The shared risk factors between atrial fibrillation and hypertension.

Risk Factor	Atrial Fibrillation	Hypertension
Increased Age	Increased risk	Increased risk
Obesity	Increased risk	Increased risk
Diabetes Mellitus	Increased risk	Increased risk
Smoking	Increased risk	Increased risk
Excess Alcohol	Increased risk	Increased risk
Sedentary Lifestyle	Increased risk	Increased risk
Obstructive Sleep Apnoea	Increased risk	Increased risk
Chronic Kidney Disease	Increased risk	Increased risk
Positive family History	Increased risk	Increased risk
Hyperthyroidism	Increased risk	Possible association
Stress	Increased risk	Increased risk
Dyslipidaemia	Increased risk	Increased risk

## Data Availability

The data relating to this study are available upon reasonable request from the corresponding author.
